# Electrochemical polarization analysis for optimization of external operation parameters in zinc fuel cells

**DOI:** 10.1039/d0ra04454g

**Published:** 2020-08-04

**Authors:** Thangavel Sangeetha, Wei-Mon Yan, Po-Tuan Chen, Cheng-Jung Yang, K. David Huang

**Affiliations:** Department of Energy and Refrigerating Air-Conditioning Engineering, National Taipei University of Technology Taipei 10608 Taiwan; Research Center of Energy Conservation for New Generation of Residential, Commercial, and Industrial Sectors, National Taipei University of Technology Taipei 10608 Taiwan; Department of Vehicle Engineering, National Taipei University of Technology Taipei Taiwan r92222019@ntu.edu.tw kdhuang@ntut.edu.tw; Program in Interdisciplinary Studies, National Sun Yat-sen University Kaohsiung 80424 Taiwan benyang0521@gmail.com

## Abstract

Zinc–air flow fuel cells utilizing zinc particles as fuel possess the potential to evolve as efficient distributed grid generators. In this research study, electrochemical impedance analysis was employed to determine the optimum design and operational parameters for the feasible maneuver and enhanced energy generation from zinc fuel cells. Polarization resistance (*R*_p_), ohmic resistance (*R*_s_), and mass transfer resistance (*R*_m_) were used as the indicators for determination of the optimum parameters of fuel cell performance. Experimental conditions optimized from previous studies like potassium hydroxide electrolyte with temperature of 25 °C and concentration of 40 wt% zinc powder quantity of 20 g, electrode reaction surface area of 48 cm^2^ were followed in the fuel cells used in the present study. Parameters like collector plate material, air flow velocity and cell operating temperature were augmented and finally were all implemented in the fuel cell and operated. Plain nickel or nickel-plated copper were both advantageous as collector plate materials whereas an air flow velocity ranging from 1–3 m s^−1^ and a cell operating temperature of 25 °C to 45 °C were beneficial for the stability and performance of the zinc fuel cells. Finally, based on the optimized parameters obtained from the above experiments, performance tests of zinc fuel cells were investigated. The maximum power produced was 16.5 W, along with a corresponding voltage of 0.8 V, maximum current density of 430 mA cm^−2^ and peak power density of 364.6 mW cm^−2^. Thus it can be concluded that the fuel cells designed and operated in this study were capable for feasible and efficient future applications.

## Abstract 2

Zinc–air flow fuel cells utilizing zinc particles as fuel possess the potential to evolve as efficient distributed grid generators.

## Graphical abstract

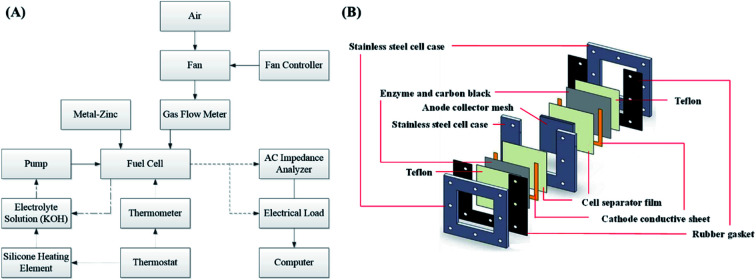

## 1 Introduction

The rocketing energy crisis and increasing environmental issues have emphasized the changeover from non-renewable to renewable energy and prerequisite research works regarding alternative sustainable energy sources. Fuel cells are considered to be the perfect choices for clean energy production as they convert chemical energy from fuels into electricity through a series of electrochemical reactions. Amongst them, zinc (Zn) fuel cells have innumerable advantages like (i) raw material (Zn) availability in abundance (ii) unwavering performance; (iii) eco-friendly nature (iv) effective energy storage and utilization. Zinc fuel cells generate electricity through the oxidation of zinc and are classified as a green renewable energy source.^1,2^ These fuel cells have undergone immense advancements and optimizations in various fields like electrode materials,^3^ zinc oxide treatments,^4^ energy storage^5^ and operational parameters.^6^ Category of the anode fuel used is a vital element in a fuel cell. Flowing particle zinc anode fuel cell systems have considerable potential for more development and feasible applications because they have larger reaction sites than systems using a fixed zinc plate as an electrode and have the advantages of rapid removal of zinc oxide and continuous replenishment of zinc particles “in electrochemical reactions” or “in electrochemical cells”.^7^ Some advantages are that the particles have a high proportion of active surface for collision with electrolyte molecules and this enhances the reaction kinetics, particle anode with flowing electrolyte may subdue hydrogen evolution reaction (HER), the zinc oxide (ZnO) product will be carried away by the electrolyte thus preventing corrosion to maintain reaction equilibrium.^8^ Arenas *et al.*^9^ have proposed a cell frame with a zinc cell anode and a cathode on the side of the frame. Zinc particles are added from the pores in the frame; the current is generated when the particles pass through a nickel mesh, and power generation is maintained by repeated addition of zinc particles and electrolyte solution. In addition, many studies have confirmed the benefits of using a flowing zinc particle system. Zn particles can promote the full migration of hydroxide ions during the battery charge/discharge process and the particles retarded the formation of dendrite in fuel cells and consequently improved the cell efficiency.^10,11^

Cheng *et al.*^12^ reviewed the advantages of a fuel cell stack employing an electrolyte flow over a single non-flow electrolyte fuel cell and found that the electrolyte flow through the reactor could reduce heat and concentration losses and improve battery performance. The properties of four major types of redox flow batteries employing zinc electrodes, including zinc–bromine, zinc–cerium, zinc–air, and zinc–nickel were investigated along with electrolyte and electrode and cell reactions for possible future advances.^13^ The management of electrochemical reaction process parameters is an important factor affecting the power generation efficiency of zinc particle cells, and this has been verified by the results of many research groups. Brillas *et al.*^14^ observed that at low temperatures, the polarization speed of the cell increases due to the reduced diffusion of electrolyte ions; conversely, battery performance is improved at high temperatures. However, the electrolyte solution tends to dry out when the temperature is overly high. Further studies where carried out where the electrolyte and reaction products were extracted from electrolytic cells for filtration, after which a fresh electrolyte is recycled back into the electrolytic cell to increase the reaction efficiency. Chen *et al.*^15^ used the method of controlling the flow rate to form a solid zinc oxide (ZnO) passivation layer by the discharge reaction and removed it prior to the charging process. In a study with a metal alkaline fuel cell employing an aluminum anode, it was reported that changing the KOH concentration increased the internal resistance of ion exchange between the two electrodes, and that increasing the concentration was beneficial for conductivity.^16^ In addition, many studies have analyzed the effects of the electrolyte concentration, temperature, and flow rate on the morphology of zinc deposits.^17–19^ The outcomes of these studies exemplified the fact that the correlation between the operating parameters of zinc fuel cells and further investigation of their optimum operation and management will be a vital and effective endeavor for enhancing the fuel cell performance.

Polarization cures are used to determine the operative conditions of a fuel cell under external resistance. They analyzed to identify four important losses such as (i) fuel crossover losses which are due to fuel depletion in the electrolyte; (ii) activation losses arising from slow reaction kinetics on the electrode surfaces (iii) ohmic losses as a result of resistance for electron and ion flow through electrodes and electrolytes and (iv) finally, concentration losses due to the reactant concentration variations on the electrode surfaces. The quantitative characteristics provided by the polarization curve can be used to determine the performance of fuel cells; however, the internal conditions cannot be effectively distinguished.^20^ Another appropriate and powerful technique for estimating the internal condition of a fuel cell is electrochemical impedance spectroscopy (EIS), also known as the AC impedance technique. The principle involves applying a small amplitude sine wave voltage/current to the electrode using an instrument and measuring the current/voltage at the response electrode. The resistance (*R*) is calculated by applying a direct current potential (current) to the circuit and measuring the current (voltage) thus generated.^21^ Although EIS and resistance are conceptually similar in that they measure the impedance of the current flow in a system, EIS is capable to accommodate time or frequency variables. Nevertheless, resistance is a physical quantity and is less effective compared to EIS. Therefore, the physical and circuit diagrams of the cell impedance model can be obtained through the equivalent circuit components.^22^ Thus we have attempted to employ EIS for the investigation of the electrochemical reaction mechanisms of zinc fuel cells in this study. The parameters investigated include the material of two collector plates, the air flow rate, and the operating temperature of the cell. First, the ohmic resistance, polarization resistance, and mass transfer resistance between the components are analyzed. Next, Nyquist plots are used to investigate each electrochemical impedance distribution and the dominant factors affecting the cell characteristics. Finally, the optimal operating performance of the cell is established.

## 2 Materials and methods

### 2.1 Cell system and experimental methods

**Fig. 1:**
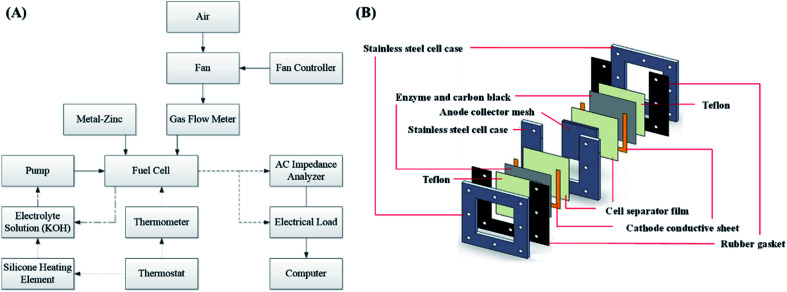
(A) Framework of the experimental platform and (B) structure of the zinc fuel cell.

**Fig. 2:**
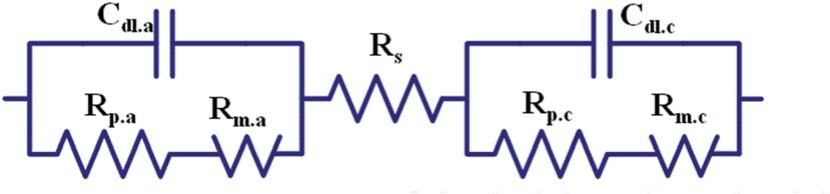
Zinc metal fuel cell component impedance.

The experimental platform consisted of a zinc fuel cell, an electrical load, an AC impedance analyzer, a portable gas flow meter, a fan, an electrolyte solution storage tank, a silicone heating element, a thermometer, a peristaltic pump, and a monitoring computer. Before the experiment commencement, the zinc fuel was positioned in the cell, the potassium hydroxide electrolyte solution was placed in the storage tank, and the electrolyte solution was heated using the thermostat and the silicone heating element. Next, the electrolyte solution was pumped into the cell using a pump, after which it returned to the storage tank through the outlet line. The air flow rate required for the experiment was provided by the fan, fan controller, and gas flow meter. The framework of the experimental platform is displayed in Fig. 1(a). Fig. 1(b) illustrates the internal structure of a zinc fuel cell. The cell case was made of stainless steel as it is advantageous for frequent disassembly and assembly and for resistance to corrosion by strong alkali. The cathode air electrode sheet was manufactured by heat-pressing of Teflon, foamed nickel, carbon black, and a catalyst. In addition to serving as a waterproof and breathable layer, Teflon can also be used to prevent the electrolyte solution from entering the cell interior and to allow oxygen in the air to react with the catalyst. Carbon black was employed as the catalyst carrier and it facilitated the uniform diffusion of gases. The other side of the cathode was a cell separator film that prevented the cathode from encountering with the anode and thus hindering internal resistance occurrence. The specifications of the instruments and reagents used in this experiment are summarized in Table 1. A computer was used to control the electrical load for current discharge, and the cell impedance was simultaneously measured using an AC impedance analyzer. The electrochemical behavior between the electrolyte and the electrode interface was simulated using an equivalent circuit formed by the circuit elements. The equivalent circuit of the impedance of the zinc fuel cell components is shown in Fig. 2. The equivalent circuit consists of two resistors (*R*) in series, in parallel with a capacitor (*C*), a Warburg element, and an ohmic resistor. When the AC frequency was very high, the internal charge distribution of the cell will be uneven, and the impedance value obtained was referred as the polarization resistance (*R*_p_). When the AC frequency was reduced, the mass transport resistance (*R*_m_) value was measured. The electrical double layer (Helmholtz model) of the electrode surface was simulated using the double-layer capacitance (*C*_dl_). The impedance of the electrolyte to ion conduction can be represented as the ohmic resistance (*R*_s_). The two parallel elements were analogous to the activation kinetics of the anode and cathode; the Warburg element was analogous to the cathode–anode mass transfer effect, and the ohmic resistance was analogous to the ohmic loss. The equivalent circuit of the cell system was represented by the vector coordinate axis in response to the impedance value at different frequencies and is divided into a real horizontal axis (*Z*′) and an imaginary vertical axis (*Z*′′). Finally, the impedance values obtained at all the different frequencies were combined to obtain a Nyquist plot, which was further used to determine all the resistance values.

**Table 1:** Experimental equipment and test reagents

Name	Model specification
Electrical load	PLZ-4W-664WA (0–150 V, 132 A, 660 W)
AC impedance analyzer	KFM2150 (660-01A)
Magnetic stirrer	HP220
Electronic precision scale	TB-600
Peristaltic pump	KS-PVP1
Thermostat	PT100
Silicone heating element	10 cm × 20 cm
Teflon thermocouple	5 cm
Nickel plate	7 cm × 7.5 cm × 0.1 cm
Nickel sheets	30 cm × 50 cm × 0.1 cm
Stainless steel mesh	140 grid per cm^2^
Copper sheets	30 cm × 50 cm × 0.1 cm
Zinc powder	45 μm
Potassium hydroxide	12 M

### 2.2 Design of the electrochemical cell experimental parameters

The collector plate material, the air flow rate, and the cell operating temperature were the experimental parameters analyzed in this study. The analyses of the anode and cathode collector plate materials were prioritized as they influenced the cell performance under the action of a strong alkaline electrolyte solution over a long period of time. After the collector material was analyzed, the air electrode was tested at different air flow rates and cell operating temperatures. The electrolyte solution (KOH) possessed a strong penetrating power and consequently it was essential to check for any leakage preceding to each experiment. Subsequently, a new separator was immersed in the electrolyte solution of an assembled cell for a period of time, and the current was observed to ascertain the stability of the separator. When the separator reached a steady state, the new air electrode sheet was subjected to 8–12 activation discharge to facilitate stability of the air electrode sheet. For all the subsequent experiments in the following sections, similar experimental conditions of 25 °C cell electrolyte solution, 40 wt% electrolyte concentration (KOH), 20 g zinc powder, 48 cm^2^ reaction surface area, 5% AC current amplitude, and a 20 kHz to 0.01 Hz frequency sweep were set except some parameters as variables in the predetermined analysis. Increasing the discharge current during the cell discharge intensified the electrochemical reaction, resulting in a relative increase in zinc fuel and air oxygen demand. To determine the change in internal resistance under different discharge conditions, experiments with discharge current densities of 10, 50, 100, 150, 200, 250, 300, 350, and 400 mA cm^−2^ were conducted in this study. The impedance of the internal resistance of the cell was measured using an AC impedance analyzer, and the effects on the anode and cathode during each interval were analyzed.

The anode was made of nickel, which had extraordinary corrosion resistance and high conductivity as the metal collector plate that served as the conductive material should be resistant to KOH corrosion.^7^ The discharge test was conducted, and the results using nickel-plated stainless steel and pure nickel were compared. With respect to the cathode, the air electrode that employed the catalyst and carbon black as the carrier was not ideal by itself. Therefore, the coverage of the metallic copper sheet around the air electrode was increased in this study. However, the copper sheet was prone to corrosion, which further augmented the impedance. Therefore, the surface was plated with nickel and compared with a pure nickel sheet during the discharge tests. Throughout the chemical reactions in the cell, the air electrode sheet impacted the reaction rate of the entire cell. This is considered to be the major bottleneck in fuel cells that use air electrode sheets. In this study, an increased amount of air was supplied through the diffusion layer to the catalyst layer for electrochemical reaction by changing the air flow rate, which increased the discharge efficiency of the cell. Then, the discharge performance of the cell was tested at air flow rates of 0, 1, 2, 3, 4, and 5 m s^−1^, and the main factors of the cell impedance were determined from the AC impedance. Since fluctuations in the cell operating temperature affected the activity between the layers, the temperature was set to 25, 35, 45, and 55 °C to measure the effect of different temperatures on the cell reaction. A thermostat was utilized to control the silicone heating element that heated the electrolyte solution in a beaker, and a pump was used to supply the electrolyte solution into the cell in a cyclic manner. The measurement of the *I*–*V* discharge curve started when the temperature of the flowing electrolyte solution inside the cell reached the experimental set temperature. The impedance of the internal resistance at different cell operating temperatures was measured using an AC impedance analyzer with a frequency sweep to analyze the main factors causing the cell impedance.

## 3 Results

### 3.1 Determination of electrochemical impedance spectra regions

**Fig. 3:**
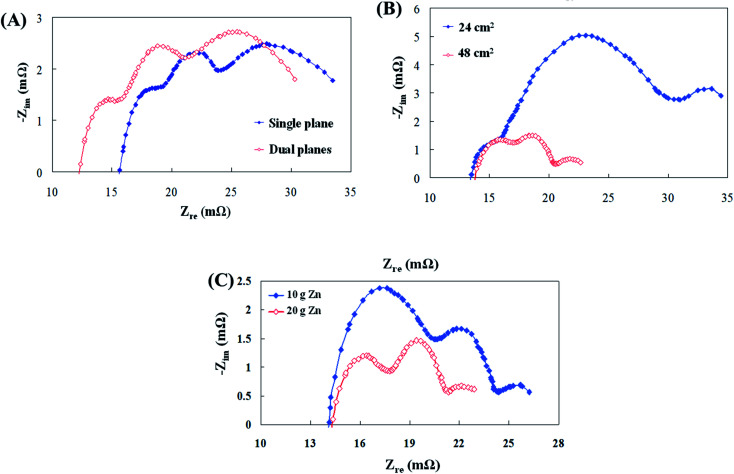
(A) AC impedance spectra for the single and dual collector plates, (B) AC impedance spectra for the cathode reaction surface area and (C) AC impedance spectra for the 10 g and 20 g zinc particles.

To resolve the electrochemical impedance spectra region, the number of collector plates, the air electrode reaction surface area, and the quantity of zinc particles were changed and analyzed in this study. Initially, as revealed in Fig. 3(a), the electrochemical impedance curve using the two collector plates is clearly shifted to the left. The equivalent circuit analysis showed that the ohmic resistance (*R*_s_) of the dual collector plates was reduced. The reason is that the zinc particles have a high surface area for contact, which might have effectively reduced the impedance of electrons during conduction and improved the discharge performance of the cell.^23^ The polarization resistance (*R*_p_) and mass transfer resistance (*R*_m_) values were noticed to have not underwent any significant change; thus, the ohmic polarization was the main influencing factor for this variable. Following that, air electrode reaction surface areas of 48 cm^2^ and 24 cm^2^ at the cathode end were subjected to AC impedance analysis. Fig. 3(b) illustrates that the second semicircular curve increased when the reaction surface area was halved. The analysis of the equivalent circuit shows that the cathodic polarization resistance (*R*_p.c_) was what affected the second semicircular curve. The reason for this increase in resistance might be due to oxygen penetration through the air electrode to the interior of the cell, which may have involved in decreasing the surface area for chemical reaction, thus resulting in an increase in impedance and a decrease in the cell discharge performance.^6,15^ Finally, 10 g and 20 g of zinc particles at the anode end were subjected to AC impedance analysis. Fig. 3(c) displays that the first semicircular curve for the 10 g zinc particles is increased. The analysis of the equivalent circuit shows that the anodic polarization resistance (*R*_p.a_) was what affected the semicircular curve. The reason for this increase in resistance was that the number of zinc particles that participated in the electrochemical reaction was less and the increased resistance caused the discharge performance of the cell to diminish. Such analysis results provided certain guidelines for battery parameter design. (1) The increase in anode contact area reduced ohmic resistance (*R*_s_) and cathodic polarization resistance (*R*_p.c_). (2) Properly increasing the quantity of zinc particles can also result in inhibited anodic polarization resistance (*R*_p.a_).

### 3.2 Flow resistance analysis

**Fig. 4:**
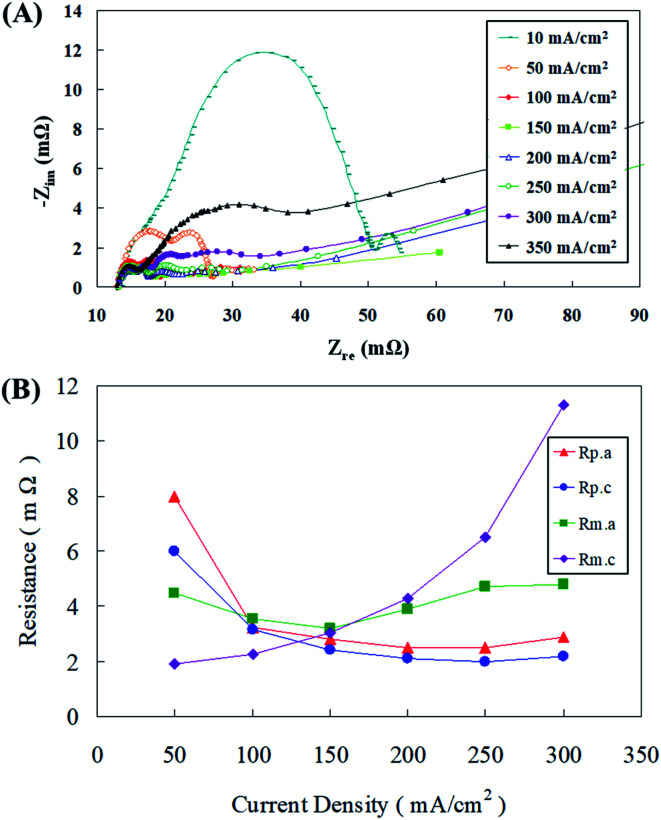
(A) AC impedance spectra and (B) impedance of the fitting equivalent circuit at different current densities.

Flow resistance analysis was conducted and Fig. 4(a and b) demonstrate the AC impedance obtained using an AC impedance analyzer at different discharge current densities. When the current density was between 10 mA cm^−2^ and 100 mA cm^−2^, the cell impedance decreased as the cell current density increased. This might be because the cell impedance will be mostly dominated by the polarization resistance at this time, of which the largest effect was that of the anode polarization resistance, followed by the cathode polarization resistance. Similar substantiations were reported by Sangeetha *et al.*,^6^ where they explained this phenomenon with Nernst equations. In a zinc fuel cell anode, the reactants are hydroxyl ions (OH^−^) and as the reactant concentration increases during fuel flow, the potential decreases, nevertheless at the cathode, it is contrary with potential reduction with reactant concentration upsurge. Ionic current output and conductivity have also been believed to play an important role in this strategy. The active impedance up surged as the current density dropped. When the current density was within the range of 100–200 mA cm^−2^, the *x*-axis cell impedance (*Z*′) was nearly unaffected by the current density afore of 30 mΩ. The cell impedance was mostly dominated by the ohmic resistance in the cell; consequently, the impedance remains at a certain value and was not affected by the cell voltage. The cell impedance gradually increased after 30 mΩ because the zinc particles were then gradually consumed during the discharge process, causing the mass transfer resistance of the anode to intensify and this effect became more pronounced as the current density increased. When the cell current density was greater than 300 mA cm^−2^, the cell impedance amplified significantly, in addition to the consumption of zinc particles. The main reason for this was that the supply of oxygen in the air was insufficient in the gas diffusion layer of the air electrode to cause an increase in the cell impedance.^24^ As the current density amplified, the dominant anode mass transfer resistance was gradually replaced by the cathode mass transfer impedance. Nevertheless, when the cathode mass transfer resistance was elevated, the anode mass transfer resistance and the cathode polarization resistance synergistically caused the polarization resistance to become indistinguishable.

The equivalent circuit module was utilized for the analyzing the Nyquist plots at each current density as shown in Fig. 4(a), the polarization resistances (*R*_p.a_ and *R*_p.c_) and mass transfer resistances (*R*_m.a_ and *R*_m.c_) at different current densities were obtained, as portrayed in Fig. 4(b). When the current density was less than 100 mA cm^−2^, the cell was dominated by the polarization resistance (*R*_p_), of which the effect of the anode polarization resistance (*R*_p.a_) was relatively high, although it decreased with the increasing current density. It was interesting to observe that when the current density was greater than 150 mA cm^−2^, the mass transfer resistance (*R*_m_) gradually became more significant. Influence of the cathode mass transfer resistance (*R*_m.c_) was the most dominant, whereas that of the anode mass transfer resistance (*R*_m.a_) was noticed to be relatively mild. In addition, it was also estimated from the mass transfer resistance curve that the mass transfer resistance (*R*_m.a_) dominated in the anode under a small current density discharge. As the current density exceeded 150 mA cm^−2^, the cathode mass transfer resistance (*R*_m.c_) gradually was overriding. Thus, the anode mass transfer resistance (*R*_m.a_) curve had a small range of variation under different discharge conditions. Such analysis results could provide some vital suggestions regarding battery parameter design that under less current densities, improving the polarization resistance (*R*_p_) can enhance battery performance. Whereas at high current densities, increasing the anode mass transfer resistance (*R*_m_), especially the cathode mass transfer resistance (*R*_m.c_), can result in effective fuel cell functioning.

### 3.3 Impedance analysis of the collector plate material

**Fig. 5:**
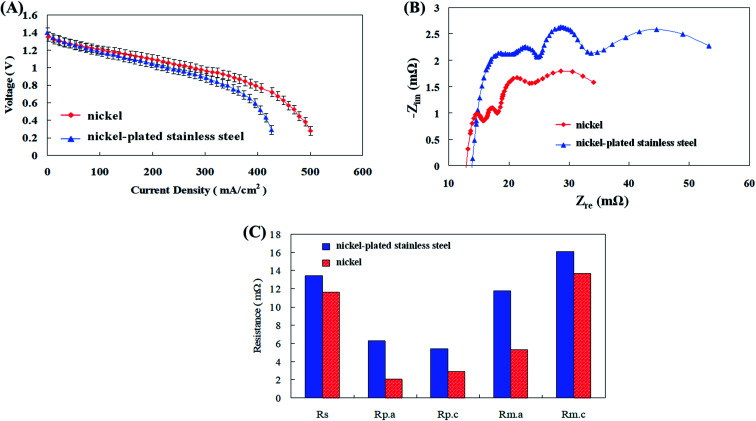
(A) *I*–*V* polarization curves, (B) AC impedance spectra, and (C) impedance of fitting equivalent circuit with different anode collector plate materials.

Anode and cathode current collectors are unavoidable and very essential components of a zinc fuel cell. Various conductive metals are employed for this purpose like nickel (Ni), stainless steel, iron, copper, silver (Ag), and gold (Au). They play a very dynamic role in the efficacious operation of the fuel cell.^25^ Anode current collectors are employed in zinc fuel cells usually to expedite the collection of electrons produced from Zn oxidation in the anode fuel. The Zn particles possess unstable and asymmetrical contact surface as this may lead to incompetent transportation of electrons to the external circuit and thus affecting the power production. Thus the electron collection is done by metal current collectors which possess effective conductivity, surface area along with stable and consistent porosity and contact surface. The better these characteristics the efficient the electron transportation will be.^26^ The release of electrons due to the oxidation of zinc particles at the anode not only causes transient conduction between particles but also causes the transfer of the electrons to the exterior by the collector plates. Although the stainless steel material used in the available anode collector plate is resistant to corrosion, it has poor conductivity. Therefore, nickel, which has a higher conductivity, was selected, and nickel-plated stainless steel plates were compared with pure nickel plates in the discharge test, and the results are shown in Fig. 5(a–c). The experimental results showed that the nickel-plated stainless steel plate had a higher impedance than the pure nickel plate. The main reason for this was that the nickel-plated stainless steel plate can cause electrons to travel primarily along the nickel surface, which had a relatively high conductivity.

However, as the discharge current increased, electrons were still conducted through the stainless steel in the interior so that the cell impedance was maintained high. The reports of Caldeira *et al.*,^27^ have supported the results of this study. Advance collectors like three dimensional plates of stainless steel and nickel were compared for use as anode current collectors in fuel cells. They observed that though stainless steel was superior to nickel in corrosion resistant capabilities, it still lacked an improved discharge and high conductivity than nickel plates. Ni anode collector plates were compared with many other metals by Zhu *et al.*^28^ Initially voltage, power and current density values of 0.7 V, 1 A cm^−2^, and 357 mW m^−2^ were produced by the fuel cells with gold current collectors. Later when they were replaced by Ni, the power performance raised almost 2 fold to 1.1 V, 2.5 A cm^−2^, and 801 mW m^−2^. They justified that the improved performance of Ni was due to its enhanced catalytic properties which might have optimistically influenced efficient ionic transport and reaction kinetics along with increased ORR and HER reaction sites for enhanced power performance of the fuel cell. Consequently, with agreement to the above mentioned substantiations, pure nickel was used as the anode collector plate material in the fuel cells of this study to improve the discharge performance.

**Fig. 6:**
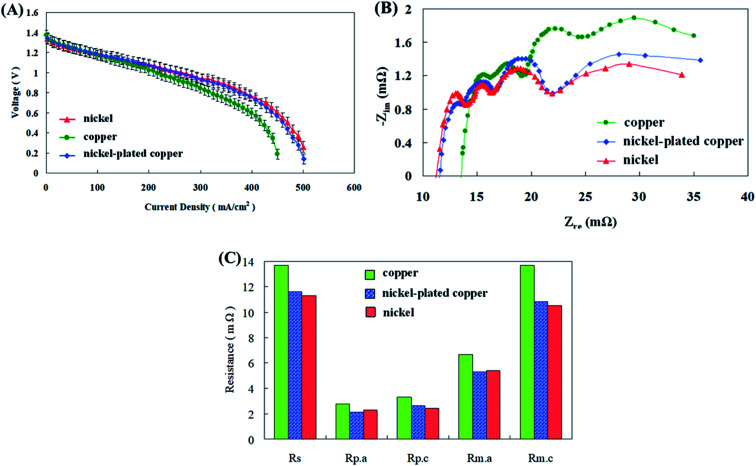
(A) *I*–*V* polarization curves, (B) AC impedance spectra, and (C) impedance of fitting equivalent circuit with different cathode collector plate materials.

Conventional cathode collector plates are made of a copper sheet with good conductivity. However, the copper sheet corrosion occurred when it was used for long periods of time, thereby increasing impedance and reducing cell performance. Sangeetha *et al.*^29^ employed copper and stainless steel sheets as cathode current collectors in zinc air fuel cells. Copper was noticed to be better than steel with increased voltage and current density production results. Copper was capable of reducing the ohmic overvoltage throughout discharge and along with that it also augmented the electron conduction efficiency of the cathode, thus amplifying the voltage and current density production. Therefore, comparative discharge tests were performed using copper, surface plated copper with nickel for corrosion resistance and a pure nickel sheet and the results are shown in Fig. 6(a–c). Copper sheet exhibited a decrease in cell performance due to increases in all impedances associated with the corrosion. The nickel-plated copper surface exhibited better discharge performance than the copper sheet due to its anti-corrosion effect. Analogous suggestions were conveyed by Kandhasamy *et al.*^30^ have designed and implemented Ni based cathode current collectors for their fuel cells. The Ni collector aided in boosted voltage and current density of the fuel cells as per the impedance results. They described that the noteworthy cause for this was the incomparable and exceptional conductivity properties and outstanding ORR activity displayed by the collector. In the present study the impedance between the nickel-plated copper surface and the nickel sheet did not differ considerably and there was no difference in the discharge performance of the cell.

**Fig. 7:**
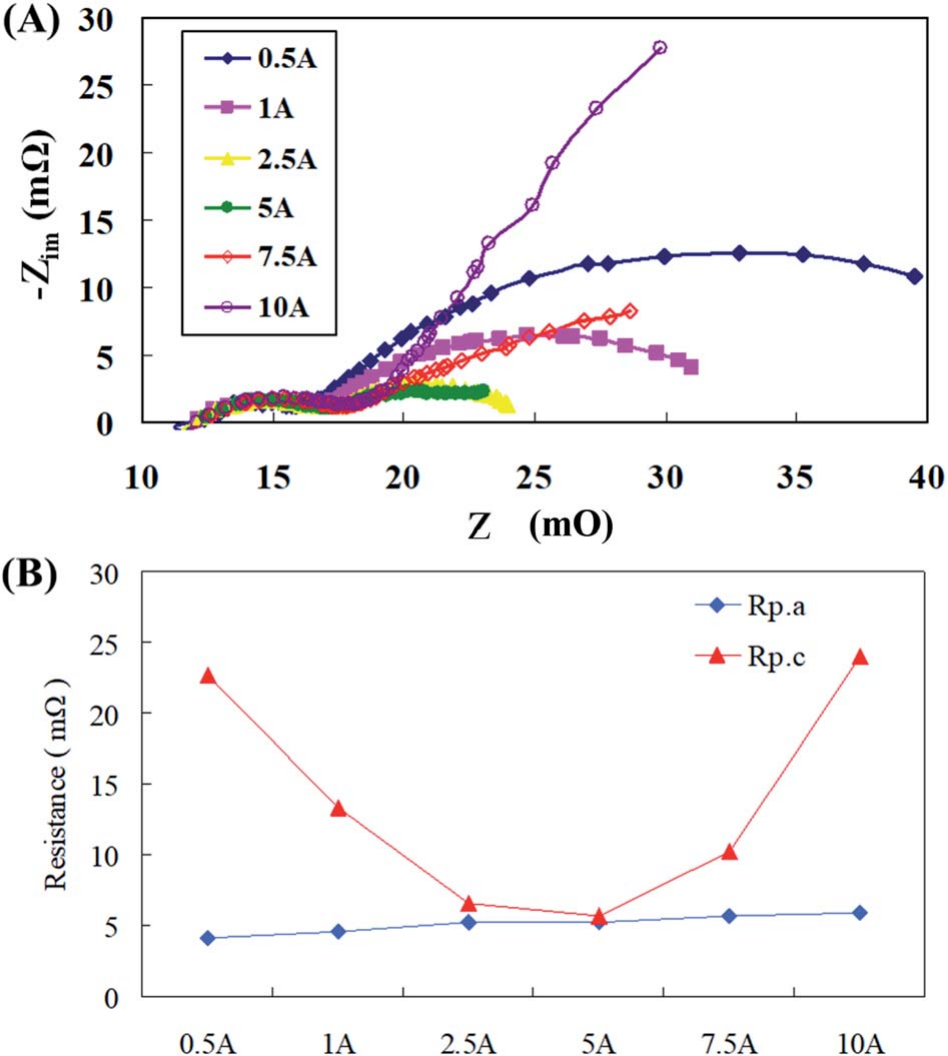
(A) AC impedance spectra and (B) impedance of fitting equivalent circuit of collector plate materials.

During material testing of the nickel-plated copper cathode collector plate, it was found that the cell performance decreased as the operation time increased; however, the pure nickel material did not exhibit this problem. To determine the reason, an AC impedance test was performed on the nickel-plated copper cathode; the results are shown in Fig. 7(a). The polarization resistance (*R*_p_) was obtained for analysis, and the results are shown in Fig. 7(b). The experimental results exhibited that the charge-transfer resistance (*R*_p.a_) at the anode end did not change considerably, while the polarization resistance (*R*_p.c_) at the cathode end increased sharply after the current increased to 10 A, resulting in a decrease in the cell performance. The reason for this is that the nickel plating of the copper sheet was performed through nickel plating of electrodes. In this process, 3–7% phosphorus was added; therefore, some substances may have been precipitated during the high-current discharge reactions in the cell, thereby damaging the air electrode and increasing the polarization resistance (*R*_p.c_) of the cathode, resulting in a decrease in the cell discharge performance.

### 3.4 Impedance analysis of air flow velocity

**Fig. 8:**
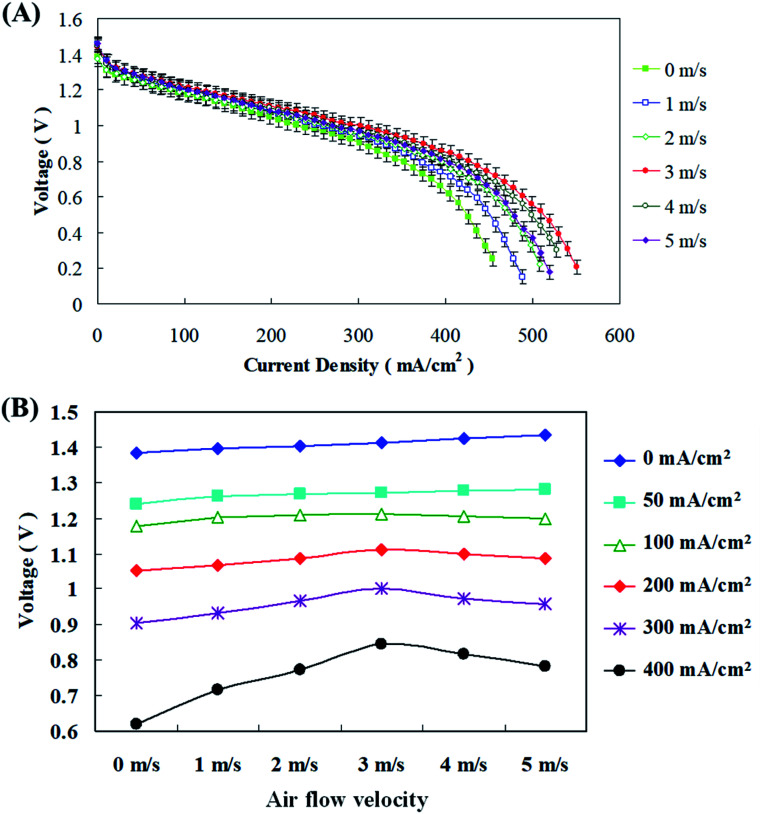
(A) *I*–*V* polarization curve and (B) discharge current–voltage performance at different air flow velocities.

Zinc fuel cells oxidize the Zn metal in the anode fuel along with atmospheric air for reduction through oxygen reduction reactions (ORR) for energy production. Thus it is essential to improve the fuel cell performance and production by air flow management. Blower and suction fans were employed for air flow into the fuel cells and their air-velocity was optimized by Sangeetha *et al.*^29^ They indicated that it is reasonable and economical to supply air to the fuel cells instead of pure oxygen. It was reported that velocities ranging from 1–3 m s^−1^ were ideal for their fuel cells as this increased the reaction kinetics of electrochemical reactions and may also enhance oxygen consumption at optimal air supply velocities. They observed that when the air velocity was from 1–3 m s^−1^ both voltage and discharge current values showed an increasing trend, but when the velocity was further increased to 4 m s^−1^, rapid downfall in power generation was witnessed. Nevertheless, cumulative cathode inlet velocities can escalate oxygen inlet as more oxygen was provided for the ORR reactions. In this study velocities of 0–5 m s^−1^ was supplied to the fuel cells and Fig. 8(a) shows that when the current density is less than 100 mA cm^−2^, different air flow rates have little effect on the cell voltage; however, the difference in voltage becomes more pronounced as the current density increased. Fig. 8(b) displays that an air flow velocity of 3 m s^−1^, instead of the maximum flow velocity of 5 m s^−1^, is optimal for operation. Therefore, if the amount of air entering the air electrode was properly increased during the zinc fuel cell discharge process, more oxygen could be effectively supplied through the diffusion layer to the catalyst layer for reaction, thereby improving the discharge performance of the cell. Further sustenance was provided by Wang *et al.*,^31^ where they interestingly noticed that as the cathode inlet air velocities amplified, the voltage production along with discharging current values also improved. This positively influenced the fuel cell performance and was definitely due to the increased electrochemical reaction rates and boosted oxygen utilization at greater air velocities. Since increasing cathode inlet velocities can increase the oxygen inlet velocities, more oxygen was provided for the ORR reactions. They also explicated that as the discharging current values were getting increased voltage production regularly reduced along with increasing air velocities. This might be due to excess water loss on the electrode which also resulted in drying of the air electrode.

**Fig. 9:**
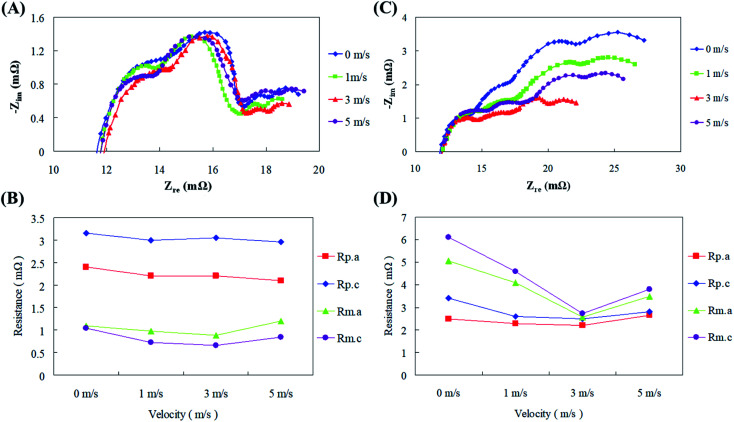
(A) AC impedance spectra at different air flow velocities with low current discharge; (B) impedance of fitting equivalent circuit at different air flow velocities with low current discharge; (C) AC impedance spectra at different air flow velocities with high current discharge; (D) impedance of fitting equivalent circuit at different air flow velocities with high current discharge.

To further understand the relationship between the air flow velocity, impedance, and current, electrochemical impedance experiments were performed independently with low (100 mA cm^−2^) and high (300 mA cm^−2^) current densities at different air flow velocities. The results in Fig. 9(a–d) show that the air flow rate had little effect on the resistance with the low current density discharge.

The polarization resistance (*R*_p_) of the cell was dominant, of which the cathode polarization resistance (*R*_p.c_) exerted a larger effect. With a high current density discharge, due to an increase in the amount of air required by the cell, increasing the air flow velocity resulted in an increase in the available air for reactions. Therefore, it was clear that the air flow velocity improved the mass transfer effect during the high current density discharge of the fuel cell, and the cell converted from the polarization resistance (*R*_p_) being dominant originally to the mass transfer resistance (*R*_m_) being dominant, of which the cathode mass transfer resistance (*R*_m.c_) wielded an enormous consequence. Pichler *et al.*^32^ had elucidated the association between fuel cell impedance performance and air flow velocity by plotting current–potential polarization curves for oxygen reduction and oxygen evolution reactions. First they concluded that supply of atmospheric air was feasible for their reactors than pure oxygen. Air flow velocities from 2–5 m s^−1^ to the fuel cells increased the current density and voltage production along with reduction in internal resistance. The ohmic and activation losses had greatly reduced with increasing air velocities up to 5 m s^−1^ above which the fuel cell performance degraded. In addition, the impedance curve demonstrates the limiting value of the air flow velocity on the cell discharge effect. Excessive air flow velocity causes the cell impedance to increase due to differential pressure, causing the discharge performance of the cell to reduce. In general, air velocity and air pressure are interrelated as the air delivered into the fuel cell with a high velocity will ultimately have a high pressure, so bigger the pressure, the greater will be the power production. Nonetheless, air might be comprised of other unreactive gases which did not partake in electrochemical reactions. They might have occupied the active sites of the air electrode thus hindering the ORR and eventually reducing the fuel cell performance.^33^ Thus it was eventually decided in the current study that the fuel cell should be operated under a controlled optimal discharge state by functioning within a certain air flow velocity range.

### 3.5 Impedance analysis of the cell operating temperature

**Fig. 10:**
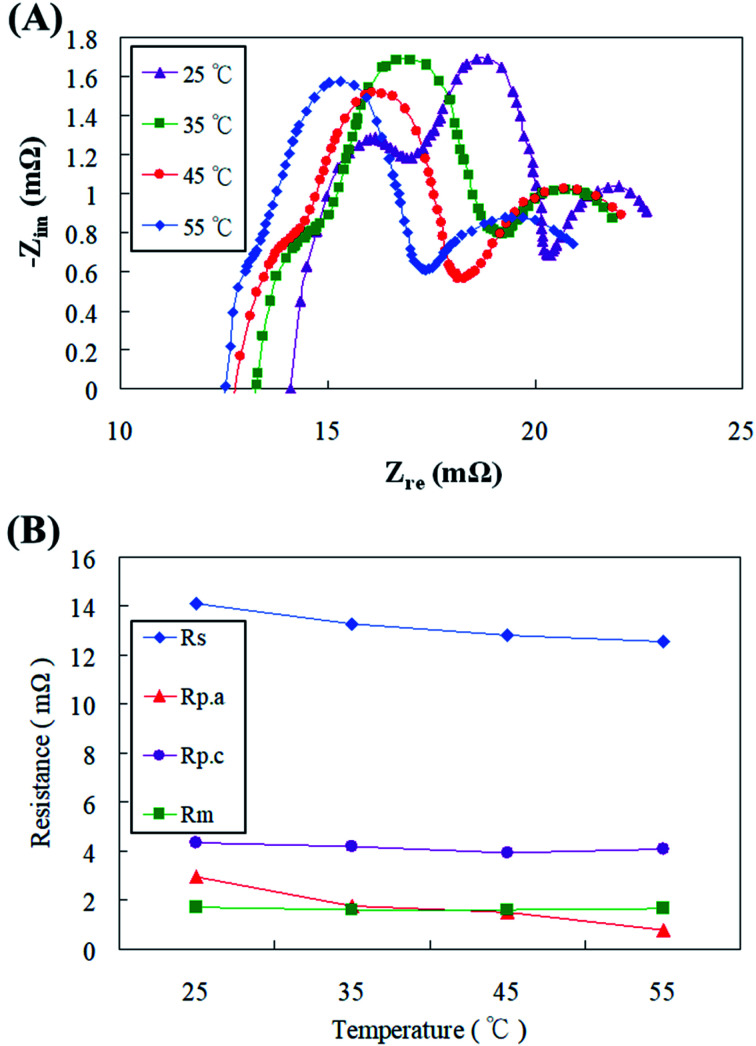
(A) AC impedance spectra and (B) impedance of fitting equivalent circuit with different cell operating temperatures.

The zinc fuel cell operating temperature has undisputable influences on various aspects like ionic transport, electrolyte behavior, zinc oxide equilibrium, dendrite deposition and reaction kinetics which would possibly alter the overall functioning of the fuel cell.^34^ Fuel cells in the present study were operated at various temperatures ranging from 25 °C to 55 °C and its effects on the fuel cell impedance behavior were investigated. Fig. 10(a) displays the AC impedance spectrum obtained at different cell temperatures with a discharge of 100 mA cm^−2^, and the impedance values are shown in Fig. 10(b). The experimental results had confirmed that increasing the cell temperature could effectively improve the ion transport of electrons and electrolyte, thereby reducing the ohmic impedance (*R*_s_). The electrochemical activity and reaction rate of the cell also amplified with temperature, which caused the polarization resistance (*R*_p_) of the cell to diminish, of which the anode polarization resistance (*R*_p.a_) was more pronounced. The reason for this is that as the temperature increases, the electrolyte ion (OH^−^) transport increases in addition to the increase in the zinc particle activity. The OH^−^ content received by the anode increases, causing the reaction potential to also increase, thereby effectively reducing the anode polarization resistance (*R*_p.a_). The reports of Gavrilović-Wohlmuther *et al.*^35^ justified the assumptions of the current study. Zinc fuel cells were operated under various temperatures like 25 °C, 50 °C, and 70 °C and the its impacts on Zn plating, crystals formation and dissolution were all reconnoitered. They reported that high operating temperatures (60 to 70 °C) were more advantageous as it augmented the electrolyte conductivity, salt solubility, and electrochemical reaction kinetics. The electrolyte lost its viscosity with cumulative temperatures, which enhanced the ionic mobility of the zincate ions. This eventually led to heightened power performance producing almost 10 times higher current density than that at low temperatures (25–50 °C). Sangeetha *et al.*^6^ had also testified zinc fuel cells under various operating parameters and one was cell operational temperature (28 to 65 °C). The voltage production was perceived to increase with increasing cell temperature with a maximum of 1.42 V produced at 50 °C due to enhanced chemical reactions and activity.

Although the air electrode of the cathode increased the activity of the catalyst due to the temperature increase, the higher the temperature, the faster the dispersion of the water in the electrolyte. This caused the OH^−^ concentration to increase, and therefore the cathode polarization resistance (*R*_p.c_) and the mass transfer resistance (*R*_m_) did not decrease. Stamm *et al.*^36^ have justified similar results where fuel cells operated under temperatures higher than 60 °C endured electrolyte vaporization which resulted in reduced ion transfer, increased product and decreased reactant concentrations and eventually abridged power performance. The zincate ion formation and deposition on the ZAFC electrodes were observed to be different when the temperature was varied. High temperatures might lead to spongy dendrite formation of zincate which possessed shoddier attachment on the electrodes, low solubility and were prone to wash out after the discharge processes.^28^ Consequently, it was resolved in the present study that the increase in the reaction temperature till 45 °C was endurable by the fuel cell while further increase might result in decreased fuel cell stability and consistency, and it was not beneficial to increase the fuel cell temperature incessantly.

### 3.6 Performance testing of the fuel cell system

**Fig. 11:**
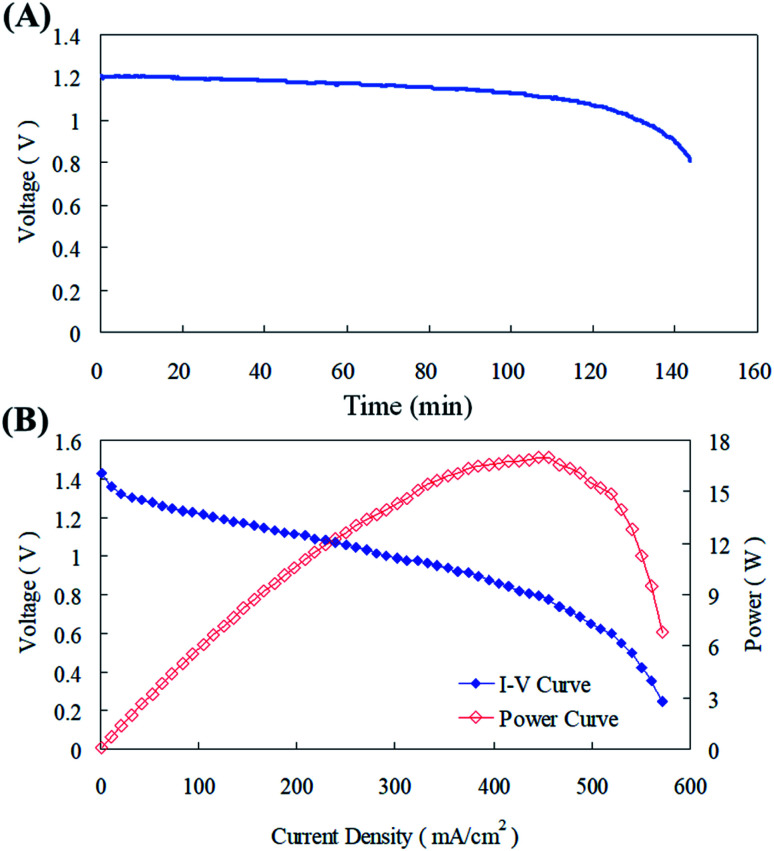
(A) Constant current discharge at 100 mA cm^2^; (B) *I*–*V* and power curves of the cell system.

The parameters optimized from this current study were implemented in the zinc fuel cell and it was operated for scrutinizing its performance.^37^ The cell system was discharged at a current of 100 mA cm^−2^, the termination voltage was set to 0.8 V, and the discharge time of the cell was measured at a fixed current using 20 g of zinc particles. Fig. 11(a) illustrates that the cell voltage is less than 0.8 V after about 140 min, and the energy density is about 540 W h kg^−1^. After disassembling the cell, some zinc particles were still found inside and the reason for this might be that after the zinc particles reacted and dissolved, the remaining particles could not effectively supply the discharge current required. Along with this the contact area with the collector plate could have reduced and this might have resulted in the inefficiency of the cell in maintaining the voltage production. The power curve in Fig. 11(b) displays that the maximum power of the cell was about 16.5 W, the current density was 430 mA cm^−2^, and the maximum power corresponded to a voltage of about 0.8 V.

## 4 Conclusions

Electrochemical impedance analysis was significantly employed for the optimization of some important operational parameters like collector plate material, air flow velocity and cell operational temperature in zinc fuel cells. The following conclusions were drawn from the study.

(1) Previously optimized parameters like potassium hydroxide electrolyte with temperature of 25 °C and concentration of 40 wt% zinc powder quantity of 20 g, electrodes reaction surface area of 48 cm^2^ were charted and employed in the present study fuel cells.

(2) Design and operational factors like collector plate material, air flow velocity and cell operating temperature were chosen for augmentation in the current study. Plain nickel or nickel plated copper were both advantageous as collector plate materials whereas an air flow velocity ranging from 1–3 m s^−1^ and a cell operating temperature of 25 °C to 45 °C were beneficial for the stability and performance of the zinc fuel cells.

(3) All the optimized variables were implemented in the fuel cells and investigated finally. The maximum power produced was 16.5 W, along with a corresponding voltage of 0.8 V, and maximum current density was 430 mA cm^−2^.

(4) Providing suitable electron reaction surface areas and appropriate air flow velocity for the electrochemical reactions will effectively result in the reduction of the overall internal resistance of the system.

(5) Finally, an increased cell operating temperature helps to improve the system performance; however, care must be taken to find a balance between the electrolyte evaporation and air flow velocity, and an increase in temperature does not necessarily result in increased efficiency. In addition, an increased operating temperature can reduce the life time and stability of the fuel cell.

(6) Effective reductions in the polarization resistance, ohmic resistance, and mass transfer resistance of the cell were observed which thereby plummeted the overvoltage failures.

(7) Therefore, the zinc fuel cell system design can reduce the internal resistance by effectively altering and choosing the design and operational parameters.

## Funding information

We are greatly thankful for the generous financial support from Ministry of Science and Technology, Taiwan, under grant numbers MOST 108-2113-M-002-021-MY2, MOST 108-2622-E-020-006-CC3 and MOST 109-2221-E-027-020.

## Conflicts of interest

The authors declare no conflict of interest.

## Supplementary Material
